# Negative Impact of Comorbidity on Health-Related Quality of Life Among Patients With Stroke as Modified by Good Diet Quality

**DOI:** 10.3389/fmed.2022.836027

**Published:** 2022-05-06

**Authors:** Thu T. M. Pham, Manh-Tan Vu, Thuc C. Luong, Khue M. Pham, Lien T. K. Nguyen, Minh H. Nguyen, Binh N. Do, Hoang C. Nguyen, Tuan V. Tran, Thao T. P. Nguyen, Hoang P. Le, Cuong Q. Tran, Kien T. Nguyen, Shwu-Huey Yang, Chaur-Jong Hu, Chyi-Huey Bai, Tuyen Van Duong

**Affiliations:** ^1^School of Public Health, College of Public Health, Taipei Medical University, Taipei, Taiwan; ^2^Faculty of Public Health, Hai Phong University of Medicine and Pharmacy, Hai Phong, Vietnam; ^3^Department of Internal Medicine, Hai Phong University of Medicine and Pharmacy, Hai Phong, Vietnam; ^4^Cardiovascular Department, Viet Tiep Friendship Hospital, Hai Phong, Vietnam; ^5^Director Office, Military Hospital 103, Hanoi, Vietnam; ^6^Cardiovascular Center, Department of Cardiology, Military Hospital 103, Hanoi, Vietnam; ^7^Faculty of Public Health, Hai Phong University of Medicine and Pharmacy, Hai Phong, Vietnam; ^8^President Office, Hai Phong University of Medicine and Pharmacy, Hai Phong, Vietnam; ^9^Rehabilitation Department, Hanoi Medical University, Hanoi, Vietnam; ^10^Rehabilitation Center, Bach Mai Hospital, Hanoi, Vietnam; ^11^Rehabilitation Department, Viet Duc University Hospital, Hanoi, Vietnam; ^12^International Ph.D. Program in Medicine, College of Medicine, Taipei Medical University, Taipei, Taiwan; ^13^Department of Infectious Diseases, Vietnam Military Medical University, Hanoi, Vietnam; ^14^Division of Military Science, Military Hospital 103, Hanoi, Vietnam; ^15^Director Office, Thai Nguyen National Hospital, Thái Nguyên, Vietnam; ^16^President Office, Thai Nguyen University of Medicine and Pharmacy, Thái Nguyên, Vietnam; ^17^Department of Neurology, Thai Nguyen University of Medicine and Pharmacy, Thái Nguyên, Vietnam; ^18^Department of Clinical Pharmacy, Thai Nguyen University of Medicine and Pharmacy, Thái Nguyên, Vietnam; ^19^Health Personnel Training Institute, University of Medicine and Pharmacy, Hue University, Hue, Vietnam; ^20^Department of Internal Medicine, Hue University of Medicine and Pharmacy, Hue University, Hue, Vietnam; ^21^Director Office, Thu Duc City Health Center, Ho Chi Minh City, Vietnam; ^22^Faculty of Health Sciences, Mekong University, Vl̃nh Long, Vietnam; ^23^Department of Health Promotion, Faculty of Social and Behavioral Sciences, Hanoi University of Public Health, Hanoi, Vietnam; ^24^School of Nutrition and Health Sciences, Taipei Medical University, Taipei, Taiwan; ^25^Nutrition Research Center, Taipei Medical University Hospital, Taipei, Taiwan; ^26^Research Center of Geriatric Nutrition, Taipei Medical University, Taipei, Taiwan; ^27^Department of Neurology, School of Medicine, College of Medicine, Taipei Medical University, Taipei, Taiwan; ^28^Department of Neurology, Taipei Medical University Shuang Ho Hospital, New Taipei City, Taiwan; ^29^Department of Public Health, College of Medicine, Taipei Medical University, Taipei, Taiwan

**Keywords:** stroke, comorbidity, diet, modification, health-related quality of life, aging

## Abstract

**Background:**

Comorbidity, along with aging, affects stroke-induced health-related quality of life (HRQoL). We examined the potential role of diet quality in modifying the association between comorbidity and HRQoL in patients with stroke.

**Methods:**

A cross-sectional study was conducted on 951 patients with stroke from December 2019 to December 2020 across Vietnam. Comorbidity was assessed using the Charlson Comorbidity Index (CCI) items and classified into two groups (none vs. one or more). Diet quality was evaluated using the Dietary Approaches to Stop Hypertension Quality (DASH-Q) questionnaire, and HRQoL was measured using the RAND-36, with a higher score indicating better diet quality or HRQoL, respectively. Besides, socio-demographics, health-related behaviors (e.g., physical activity, smoking, and drinking), disability (using WHODAS 2.0), and health literacy were also assessed. Linear regression analysis was utilized to explore the associations and interactions.

**Results:**

The proportion of patients with stroke aged ≥65 years and having comorbidity were 53.7 and 49.9%, respectively. The HRQoL scores were 44.4 ± 17.4. The diet quality was associated with higher HRQoL score (regression coefficient, B, 0.14; (95% confidence interval, 95% CI, 0.04, 0.23; *p* = 0.004), whereas comorbidity was associated with lower HRQoL score (B, −7.36; 95% CI, −9.50, −5.23; *p* < 0.001). In interaction analysis, compared to patients without comorbidity and having the lowest DASH-Q score, those with comorbidity and higher DASH-Q score had a higher HRQoL score (B, 0.21; 95% CI, 0.03, 0.39; *p* = 0.021).

**Conclusion:**

The findings showed that good diet quality could modify the adverse impact of comorbidity on HRQoL in patients with stroke. Diet quality should be considered as a strategic intervention to improve the HRQoL of patients with stroke, especially those with comorbidity, and to promote healthier aging.

## Introduction

Stroke, a common non-communicable disease (NCD), has generated an increasing burden worldwide because of high incidence, prevalence, mortality rate, and impaired health outcomes (1, 2). Health-related quality of life (HRQoL) is one of the stroke outcomes in the stroke survivors’ experience of significant impairment in HRQoL (3, 4). Moreover, a forward trend of stroke cases is predicted to increase in the 21st century along with the aging of the population, which will lead to poor health outcomes, including HRQoL (5). Hence, improving HRQoL should become a part of goal setting for stroke rehabilitation, especially in the aging population. Also, identification of the possible modifiable factors regarding HRQoL is necessary for post-stroke therapeutic strategies and health promotion.

Many predictors of HRQoL in patients with stroke were investigated, including comorbidity (6). Although the comorbid conditions are common in patients with stroke (7), the comprehensive reports on post-stroke HRQoL related to comorbidity have not been adequately studied. The reason is that the association between comorbidity and HRQoL varied across studies because of the differences in cultural backgrounds (6) and comorbidity measures (8). Also, the extent of HRQoL impairment may vary due to the Coronavirus Disease 2019 (COVID-19) crisis because the occurrence of stroke becomes more frequent with a high burden of co-existing diseases (9). In addition, stroke rehabilitation contributed to improving HRQoL (10), but most stroke rehabilitation trials did not include those with comorbidity (11), which in turn influences the HRQoL reports. Therefore, a greater understanding of the comorbidity-HRQoL relationship could optimize the care and rehabilitation among patients with stroke.

Nutrition intervention is one of the global prevention strategies against NCDs, including stroke, because incorrect nutrition was the metabolic risk factor leading to premature NCD deaths (12). In the literature, the Dietary Approaches to Stop Hypertension (DASH) dietary pattern (13) was investigated to reduce stroke occurrence, but few studies approached the diet quality (14). The infrequent use of diet quality in the previous studies was because of the ambiguous definition and inconsistency in measurement tools (15). In addition, the impact of nutrition on HRQoL in patients with stroke has not been consistent, whereas good dietary habits could improve HRQoL (16), nutrition therapy showed no effects on stroke outcomes, including HRQoL (17).

Reports on the links between comorbidity and diet quality with HRQoL were investigated but are still scarce and need to have a comprehensive sight. Therefore, our purpose was to explore the possible role of diet quality in modifying the relationship between comorbidity and HRQoL among patients with stroke.

## Materials and Methods

### Study Population

During December 2019 and December 2020, we conducted a cross-sectional study on stroke inpatients in six hospitals across Vietnam, including one hospital in the south, one in the central, and four in the north. Out of invited hospitals across Vietnam, six hospitals agreed to participate in our study because many hospitals have to re-arrange their resources and budgets for COVID-19 epidemic prevention. These six hospitals are large, with around 600–1,900 beds, and located in the big cities in Vietnam. Patients with stroke were diagnosed by neurologists and classified based on the International Classification of Disease 10th revision (ICD-10) coding I60-I69 (18). Patients were recruited consecutively in the cardiovascular, neurology, and rehabilitation departments. Since the national lockdown was implemented in Vietnam between the 1st and 22nd of April 2020 due to the COVID-19 pandemic, the recruitment was postponed. The eligible participants were those aged ≥18 years, had stable conditions of stroke (e.g., a Mini-Mental State Examination score of ≥22), with the ability to respond to questions. Besides, patients with stroke with aphasia or visual impairment and diseases affecting cognition (e.g., dementia) were excluded to ensure that patients could be able to complete a face-to-face interview. To have an adequate sample for the statistical analysis, a minimum sample size of 262 was calculated with an effect size of 0.05, type I error of 0.05, power of 0.95, and 8 predictors in the multiple linear regression using G*Power software version 3.1.9.7 (19). We recruited 951 qualified patients, which was large enough for the statistical method used in this study. All participants were asked to provide informed consent before administering the survey.

### Data Collection

The valid questionnaires in the Vietnamese language were used in the survey, including the 16-item Charlson Comorbidity Index (CCI), The Dietary Approaches to Stop Hypertension Quality questionnaire, a 36-item short-form survey (SF-36), the International Physical Activity Questionnaire, the World Health Organization Disability Assessment Schedule 2.0 (WHODAS 2.0), and a 12-item short form of the HL survey. A face-to-face interview was performed at the bedside within about 30 min to complete the survey for one patient. Interviewers were doctors, nurses, and medical students who first received a 4 h training session about data collection. Besides, interviewers also received infection control training from each hospital based on the guidelines of the Ministry of Health in Vietnam (20), and the WHO (21), including wearing a mask, washing hands, and physical distance. In addition, data were also extracted from medical records.

#### Assessment of Comorbidity

The Comorbidity was evaluated based on the 16-item CCI (22, 23). We removed two items, including cerebrovascular disease or stroke and dementia, because of reported patients and exclusive criteria, respectively. Then, the comorbidity was regrouped into none vs. one or more CCI.

#### Assessment of Diet Quality

Diet quality was assessed using the DASH-Quality (DASH-Q) questionnaire, which rates the number of days (from 0 to 7) consuming the 11 food items in the previous 7 days (24), including nuts or peanut butter; beans, peas, or lentils; eggs; pickles, olives, or other vegetables in brine; fruits and vegetables (≥5 servings); fruits (>1 serving); vegetables (>1 serving); drink milk (in a glass, with cereal, or in coffee, tea, or cocoa); broccoli, collard greens, spinach, potatoes, squash, or sweet potatoes; apples, bananas, oranges, melon, or raisins; and whole-grain bread, cereals, grits, oatmeal, or brown rice. In the Vietnamese context, the item “drink milk (in a glass, with cereal, or in coffee, tea, or cocoa)” was excluded from the survey. The DASH-Q questionnaire with ten items was validated and used in Vietnam (25). The sum score was ranked between 0 and 70, with higher DASH-Q scores reflecting a better diet quality.

#### Assessment of Health-Related Quality of Life

Health-related quality of life was evaluated using a 36-item short-form survey (SF-36). The SF-36 consists of eight domains, including general health, emotional role, physical role, physical functioning, social function, emotional well-being, pain, and energy/fatigue (26). The SF-36 was used in Vietnamese Americans (27) and Vietnamese contexts (28, 29). The scoring algorithms were mentioned in detail in the user manual (30). The SF-36 score varied from 0 (worst HRQoL) to 100 (best HRQoL).

#### Assessment of Covariates

Patients were asked about the occurrence of stroke and whether it was the first or recurrent stroke. Besides, the classification of stroke was defined using ICD-10 (18). Then, the stroke classification was regrouped into a hemorrhagic stroke, ischemic stroke (or cerebral infarction), and cerebrovascular diseases to facilitate the analysis.

Socio-demographic factors were self-reported, including age (years), gender, occupation, education attainment, ability to pay for medication, marital status, and social status. Besides, the body mass index (BMI, kg/m^2^) was calculated.

The present health-related behaviors were self-reported, including drinking (no vs. yes) and smoking (never vs. ever smoked). In addition, the level of physical activity (PA) was assessed using the International Physical Activity Questionnaire (IPAQ). Patients rated the time during the previous 7 days (number of days per week and minutes per day) spending on four activities, including vigorous, moderate, walking, and sitting (31). The IPAQ was validated and used in Vietnam (25, 32). The PA levels were calculated according to the metabolic equivalent tasks scored in minutes per week (MET-min/wk) (33). The total MET score was estimated as the sum of minutes per week of PA at different levels of vigorous, moderate, walking, and sitting multiplied by 8, 4, 3.3, and 1, respectively. The higher MET scores represented the more intensive levels of PA. Then, we categorized MET scores into tertiles to facilitate the analysis.

The disability level of patients with stroke was measured using the WHODAS 2.0 with 12 items. The WHODAS 2.0 was used in diverse cultures and all adult populations (34). Patients rated the difficulties in performing daily activities over the previous 30 days on a 5-point scale from 1 (none) to 5 (extreme or cannot do). The overall score was computed by summing 12-item scores, with the greater scores representing the higher level of disability.

With regards to patients with stroke, the health literacy (HL) index was evaluated to assess their ability to access, understand, judge, and utilize health-related information in terms of healthcare, health promotion, and disease prevention (35). A 12-item short form of the HL survey was used, which was validated and used widely in Vietnam (36, 37). Patients rated the difficult extent of each item on a 4-point Likert scale from 1 (very difficult) to 4 (very easy). The HL index was calculated using the formula:

$$I\,n\,d\,e\,x=\left(m\,e\,a\,n-1\right)\times \left(\frac{50}{3}\right)$$

Where *Index* is the standardized HL indices, *mean* is the average of 12 items, *1* is the minimal possibility of the mean, *50* is the chosen maximum HL index score, and 3 is the range of the mean. Thus, the HL index ranged from 0 to 50, and the higher scores indicated a better HL.

### Ethical Consideration

The study was reviewed and approved by the Institutional Ethical Review Committee of Hanoi School of Public Health, Vietnam (IRB Nos. 498/2019/YTCC-HD3 and 312/2020/YTCC-HD3).

### Statistical Analysis

First, we performed the descriptive analysis and used a one-way ANOVA test to compare the mean of HRQoL in different categories of independent variables (IVs). Second, we used linear regression analysis to investigate the association between CCI and DASH-Q with HRQoL. In the bivariate analysis, factors with *p* < 0.05 were selected for adjustment in the multiple analysis models. Besides, the Spearman correlation was tested to avoid multicollinearity among IVs. The results showed that occupation moderately correlated with age (*rho* = 0.35), and MET-min/wk (*rho* = −0.32); gender moderately correlated with drinking (*rho* = 0.45); WHODAS 2.0 moderately correlated with CCI (*rho* = 0.30); and MET-min/wk (*rho* = −0.32) (Supplementary Table 1). Therefore, several representative factors were selected for the multiple analysis models, including age, gender, marital status, stroke occurrence, stroke classification, CCI, DASH-Q, and HL index. Third, we performed the interaction analysis to explore the potential modification impacts of DASH-Q on the relationship between CCI and HRQoL. To visualize the results of the interaction model, we conducted a simple slope analysis using PROCESS Macro of SPSS for moderation analysis. The slope plots were drawn using the evaluated values of HRQoL for two categories of comorbidity (non-CCI vs. CCI) by three values of DASH-Q (one standard deviation below the mean; the mean; one standard deviation above the mean). Data were analyzed using IBM SPSS Version 20.0 (IBM Corp., Armonk, NY, United States). The significance level was set at a *p*-value < 0.05.

## Results

### Characteristics of Patients With Stroke

Among 951 patients with stroke, 82.5% experienced the first stroke; 67.1% were classified as ischemic stroke, followed by hemorrhagic stroke (23.2%) and stroke due to cerebrovascular diseases (9.7%); and 49.9% had comorbidity. Patients aged 65 years or above accounted for 53.7%, and 59.2% were men. The overall score of HRQoL was 44.4 ± 17.4 and significantly different in categories of age, gender, occupation, marital status, stroke occurrence, stroke classification, comorbidity, drinking, and physical activity (*p* < 0.05) (Table 1).

**TABLE 1:** Characteristics and health-related quality of life (HRQoL) in patients with stroke (*n* = 951).

Variables	Total	HRQoL	
	
	*N* (%)	Mean ± SD	*p*
Age (years)			<0.001
<65	440 (46.3)	47.1 ± 17.6	
≥65	511 (53.7)	42.0 ± 16.9	
Gender			0.013
Women	388 (40.8)	42.7 ± 16.8	
Men	563 (59.2)	45.5 ± 17.1	
Occupation			<0.001
Working	518 (54.5)	47.2 ± 17.6	
Retired or infirmity	433 (45.5)	40.9 ± 16.5	
Education attainment			0.344
Illiterate or elementary	215 (22.6)	43.1 ± 15.7	
Junior high	257 (27.1)	43.5 ± 18.3	
Senior high	251 (26.4)	45.4 ± 18.0	
College/university or higher	227 (23.9)	45.3 ± 17.4	
Ability to pay for medication			0.691
Very or fairly difficult	423 (44.5)	44.1 ± 16.6	
Very or fairly easy	528 (55.5)	44.6 ± 18.1	
Marital status			0.007
Married	837 (88.0)	448 ± 17.8	
Single or Widowed/Divorced/Separated	114 (12.0)	40.9 ± 13.8	
Social status			0.056
Low	111 (11.7)	41.4 ± 15.8	
Middle or high	840 (88.3)	44.8 ± 17.6	
BMI (kg/m^2^)			0.382
Underweight (<18.5)	90 (9.5)	41.9 ± 16.9	
Normal weight (18.5 ≤ BMI < 24)	794 (83.7)	44.6 ± 17.2	
Overweight/Obese (≥24)	65 (6.8)	44.6 ± 20.3	
Stroke occurrence			0.040
First stroke	785 (82.5)	44.9 ± 16.9	
Recurrent stroke	166 (17.5)	41.6 ± 19.5	
Stroke classification			0.031
Cerebrovascular diseases	92 (9.7)	48.9 ± 16.0	
Infarction	637 (67.1)	43.9 ± 17.8	
Hemorrhage	220 (23.2)	43.9 ± 16.6	
CCI			<0.001
None	476 (50.1)	48.1 ± 17.0	
One or more	475 (49.9)	40.6 ± 17.0	
Smoking			0.698
Never smoke	544 (57.2)	44.5 ± 17.2	
Ever smoke	407 (42.8)	44.1 ± 17.8	
Drinking			0.001
No	661 (69.5)	43.1 ± 17.5	
Yes	290 (30.5)	47.2 ± 17.0	
Physical activity (MET-min/wk)			<0.001
Tertile 1 (MET ≤ 597)	324 (34.1)	37.7 ± 15.8	
Tertile 2 (597 < MET ≤ 3726)	312 (32.8)	47.1 ± 16.7	
Tertile 3 (MET > 3726)	315 (33.1)	48.5 ± 17.6	
DASH-Q (mean ± SD)	29.2 ± 11.8		
WHODAS 2.0 (mean ± SD)	32.3 ± 13.5		
HL index (mean ± SD)	23.4 ± 10.0		
HRQoL (mean ± SD)	44.4 ± 17.4		

*SD, standard deviation; HRQoL, health-related quality of life; BMI, body mass index; CCI, Charlson Comorbidity Index; MET-min/wk, metabolic equivalent task scored in minutes per week; DASH-Q, Dietary Approaches to Stop Hypertension Quality; WHODAS 2.0, World Health Organization Disability Assessment Schedule 2.0; HL, health literacy.*

### Associated Factors of Health-Related Quality of Life in Patients With Stroke

In the multiple regression model (Table 2), patients with stroke aged ≥65 years (regression coefficient, B, −5.06; 95% confidence interval, 95% CI, −7.26, −2.86; *p* = 0.013), classified as hemorrhagic stroke (B, −5.04; 95% CI, −9.28, −0.81; *p* = 0.012), and with one or more comorbidities (B, −7.36; 95% CI, −9.50, −5.23; *p* < 0.001) had a lower score of HRQoL compared to their counterparts. Whereas, patients with one-point increment in DASH-Q and HL index had a 0.14-point increment (B, 0.14; 95% CI, 0.04, 0.23; *p* = 0.004) and a 0.36-point increment (B, 0.36; 95% CI, 0.25, 0.47; *p* < 0.001) in HRQoL, respectively.

**TABLE 2:** Associated factors of HRQoL in patients with stroke (*n* = 951).

Variables	HRQoL			
	B (95% CI)*	p*	B (95% CI)**	p**
**Age (years)**				
<65	Ref		Ref	
≥65	−5.06 (−7.26, −2.86)	<0.001	−2.78 (−4.98, −0.58)	0.013
**Gender**				
Women	Ref		Ref	
Men	2.87 (0.62, 5.12)	0.013	1.37 (−0.82, 3.56)	0.220
**Occupation**				
Working	Ref			
Retired or infirmity	−6.33 (−8.52, −4,14)	<0.001		
**Education attainment**				
Illiterate or elementary	Ref			
Junior high	0.45 (−2.71, 3.61)	0.781		
Senior high	2.32 (−0.86, 5.49)	0.153		
College/university or higher	2.25 (−1.01, 5.50)	0.176		
**Ability to pay for medication**				
Very or fairly difficult	Ref			
Very or fairly easy	0.45 (−1.78, 2.68)	0.691		
**Marital status**				
Married	Ref		Ref	
Single or Widowed/Divorced/Separated	−3.90 (−7.30, −0.49)	0.025	−2.05 (−5.44, 1.34)	0.236
**Social status**				
Low	Ref			
Middle or high	3.36 (−0.08, 6.81)	0.056		
**BMI (kg/m^2^)**				
Normal weight (18.5 ≤ BMI < 24)	Ref			
Underweight (<18.5)	−2.67 (−6.47, 1.13)	0.168		
Overweight/Obese (≥24)	0.08 (−4.33, 4.49)	0.972		
**Stroke occurrence**				
First stroke	Ref		Ref	
Recurrent stroke	−3.37 (−6.29, −0.46)	0.023	−1.52 (−4.32, 1.27)	0.285
**Stroke classification**				
Cerebrovascular diseases	Ref		Ref	
Infarction	−5.04 (−8.85, −1.24)	0.009	−2.65 (−6.24, 0.95)	0.149
Hemorrhage	−5.04 (−9.28, −0.81)	0.020	−5.16 (−9.17, −5.11)	0.012
**CCI**				
None	Ref		Ref	
One or more	−7.46 (−9.63, −5.29)	<0.001	−7.25 (−9.40, −5.11)	<0.001
**Smoking**				
Never smoked	Ref			
Ever smoked	−0.44 (−2.68, 1.80)	0.698		
**Drinking**				
No	Ref			
Yes	4.11 (1.71, 6.50)	0.001		
**Physical activity (MET-min/wk)**				
Tertile 1 (MET ≤ 597)	Ref			
Tertile 2 (597 < MET ≤ 3726)	9.45 (6.84, 12.06)	<0.001		
Tertile 3 (MET > 3726)	10.85 (8.25, 13.45)	<0.001		
DASH-Q (1-point increment)	0.22 (0.13, 0.31)	<0.001	0.14 (0.04, 0.23)	0.004
WHODAS 2.0 (1-point increment)	−0.74 (−0.81, −0.67)	<0.001		
HL index (1-point increment)	0.42 (0.31, 0.53)	<0.001	0.36 (0.25, 0.47)	<0.001

HRQoL, health-related quality of life; BMI, body mass index; CCI, Charlson Comorbidity Index; MET-min/wk, metabolic equivalent task scored in minutes per week; DASH-Q, Dietary Approaches to Stop Hypertension Quality; WHODAS 2.0, World Health Organization Disability Assessment Schedule 2.0; HL, health literacy. *Results of bivariate linear regression analysis. **Results of multivariate linear regression analysis adjusted for age, gender, marital status, stroke occurrence, stroke classification, and health literacy.

### Modified Impact by Diet Quality on the Association Between Comorbidity and Health-Related Quality of Life

**FIGURE 1:**
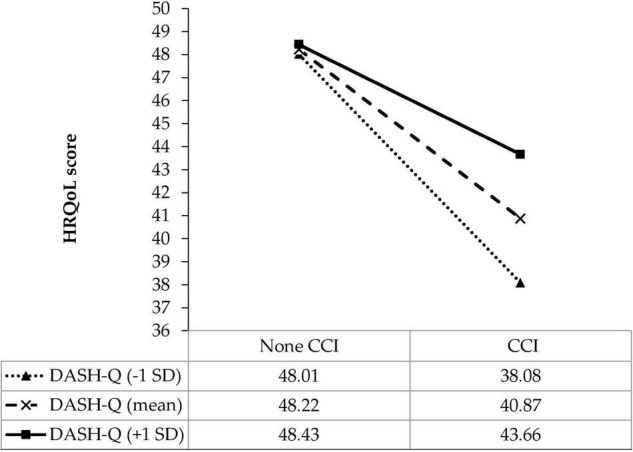
Simple slope plot for the interaction between diet quality and comorbidity on HRQoL among patients with stroke (*n* = 951). DASH-Q, diet quality; CCI, comorbidity; SD, standard deviation.

As shown in Table 3, the interaction model between comorbidity and DASH-Q was examined. The results revealed that among patients with stroke having the lowest score of DASH-Q, those with comorbidity had a lower score of HRQoL than those without comorbidity (B, −13.43; 95% CI, −19.10, −7.75; *p* < 0.001). However, patients with comorbidity and every one-point increment of DASH-Q score significantly had a 0.21-point increment of HRQoL score (B, 0.21; 95% CI, 0.03, 0.39; *p* = 0.021). Besides, the results of interaction were visualized in Figure 1. Simple slope analysis showed that the impact of comorbidity on HRQoL was weaker by higher DASH-Q values from one SD below the mean (B, −9.93; 95% CI, −12.92, −6.93; *p* < 0.001), to the mean (B, −7.35; 95% CI, −9.48, −5.22; *p* < 0.001), one SD above the mean (B, −4.77; 95% CI, −7.78, −1.77; *p* = 0.002).

**TABLE 3:** Interaction of comorbidity and diet quality on HRQoL among patients with stroke (*n* = 951).

Interaction	HRQoL			
	B (95% CI)*	p*	B (95% CI)**	p**
Non-CCI × DASH-Q (lowest score)	Ref		Ref	
CCI × DASH-Q (lowest score)	−15.62 (−21.41, −9.83)	<0.001	−13.43 (−19.10, −7.75)	<0.001
Non-CCI × DASH-Q (1-point increment)	0.03 (−0.10, 0.16)	0.676	0.03 (−0.10, 0.16)	0.651
CCI × DASH-Q (1-point increment)	0.30 (0.12, 0.49)	0.001	0.21 (0.03, 0.39)	0.021

HRQoL, health-related quality of life; CCI, Charlson Comorbidity Index; DASH-Q, Dietary Approaches to Stop Hypertension Quality. *Results of bivariate linear regression analysis. **Results of multivariate linear regression analysis adjusted for age, gender, marital status, stroke occurrence, stroke classification, and health literacy.

## Discussion

The current study emphasized the independent and interactive impacts of comorbidity and diet quality on HRQoL in patients with stroke. Comorbid conditions and poor diet quality influenced the HRQoL reduction, and good diet quality had a potential role in improving the negative impact of comorbidity on HRQoL.

In the current study, the HRQoL was impaired in aged patients with stroke and those with comorbidity. In the existing literature, the harmful impacts of comorbidity on HRQoL were reported in patients with CVDs, such as hypertension (38), atrial fibrillation (39), and stroke (40). On the one side, the prevalence of comorbidities (e.g., hypertension and diabetes) in patients with stroke increased the brain’s vulnerability to ischemic injury, contributing to worse stroke outcomes (41, 42). Moreover, comorbid conditions induced endothelial oxidative stress and peripheral inflammation (43, 44), which resulted in the loss of physical and mental health and mortality. On the other side, together with aging issues, comorbidities and stroke events become prevalent, which interferes with HRQoL (45).

A healthy diet was considered one of the cardiovascular health metrics to identify optimal brain health (46) and significantly associated with a greater HRQoL (47). Also, a higher DASH-Q score, characterizing a better diet quality, was associated with a greater HRQoL level in the present study. Furthermore, several studies shared similar findings that the higher diet quality scores were significantly associated with better HRQoL in breast cancer survivors (48) and older adults (49). Additionally, food security becomes a significant challenge during the COVID-19 pandemic (50), impacting the diet quality and influencing the HRQoL of patients with stroke. Therefore, an appropriate strategy should be developed to improve diet quality for patients with stroke, which may help to enhance their HRQoL, especially during the COVID-19 crisis.

In addition, current results indicated that good diet quality could reduce the harmful impact of comorbidity on HRQoL. This association may be explained through the role of inflammatory reaction. As comorbid health problems reduced stroke-related HRQoL because of inducing inflammatory responses, Levard et al. indicated that the lack of nutrients had a link with stroke inflammatory and immune responses (43). Therefore, a sufficient supply of nutrients through good diet quality could improve stroke inflammation and refine HRQoL.

Our study was strong in measuring the power up to 99%, which is overpowered. This power indicated a good ability to provide evidence in associations between comorbidity, diet quality, and HRQoL. However, several limitations should be considered. First, in a cross-sectional study, causality cannot be implied, such as the time order between comorbidities, stroke events, and the prognosis of HRQoL of patients; only associations were recognized. Second, several factors may be the confounders of HRQoL that were not assessed in our study, such as the time from the stroke onset to receiving treatment, length of hospital stay, and COVID-19-like symptoms. Third, the types of food included in the DASH-Q questionnaire were not comprehensive compared to dietary records, and patients may make recall mistakes while responding to the food items eaten within the past 7 days. However, the food items listed in the DASH-Q questionnaire were proper to assess the diet quality within the scope of Vietnamese patients with stroke. Fourth, the patients were not randomly selected, which may affect the generalizability of the study. The research findings should be interpreted with caution. Last, HRQoL is inherently subjective and changeable, and the measurement of HRQoL might be mutable across instruments, which might influence the results.

## Conclusion

Comorbidity and poor diet quality were predictors of worse HRQoL in patients with stroke. Notably, a good diet quality could modify the negative impact of comorbidity on HRQoL. Therefore, a healthy diet should be promoted in stroke care, especially in patients with comorbidities, as a strategic intervention to improve their HRQoL and have healthy aging.

## Data Availability Statement

The raw data supporting the conclusions of this article will be made available upon reasonable request to the corresponding authors.

## Ethics Statement

The studies involving human participants were reviewed and approved by the Institutional Ethical Review Committee of Hanoi University of Public Health, Vietnam (IRB Nos. 498/2019/YTCC-HD3 and 312/2020/YTCC-HD3). The patients/participants provided their written informed consent to participate in this study.

## Author Contributions

TP, M-TV, TL, KP, LN, MN, BD, HN, TT, HL, TN, CT, KN, S-HY, C-JH, C-HB, and TD: conceptualization, methodology, validation, investigation, data curation, and writing review and editing draft. TP, C-HB, and TD: formal analysis and writing the original draft. TP, MN, and TN: project administration. C-HB and TD: supervision and funding acquisition. All authors have read and approved the final manuscript.

## Conflict of Interest

The authors declare that the research was conducted in the absence of any commercial or financial relationships that could be construed as a potential conflict of interest.

## Publisher’s Note

All claims expressed in this article are solely those of the authors and do not necessarily represent those of their affiliated organizations, or those of the publisher, the editors and the reviewers. Any product that may be evaluated in this article, or claim that may be made by its manufacturer, is not guaranteed or endorsed by the publisher.

## References

[1] Béjot Y Daubail B Giroud M. Epidemiology of stroke and transient ischemic attacks: current knowledge and perspectives. *Rev Neurol.* (2016) 172:59–68. 10.1016/j.neurol.2015.07.013 26718592

[2] Katan M Luft A. Global burden of stroke. *Semin Neurol.* (2018) 38:208–11. 10.1055/s-0038-1649503 29791947

[3] Bello UM Chutiyami M Salihu D Abdu SI Tafida BA Jabbo AA Quality of life of stroke survivors in Africa: a systematic review and meta-analysis. *Qual Life Res.* (2021) 30:1–19. 10.1007/s11136-020-02591-6 32712933

[4] Donkor ES. Stroke in the 21*^st^* century: a snapshot of the burden, epidemiology, and quality of life. *Stroke Res Treat.* (2018) 2018:3238165. 10.1155/2018/3238165 30598741 PMC6288566

[5] Béjot Y Bailly H Durier J Giroud M. Epidemiology of stroke in Europe and trends for the 21st century. *Presse Med.* (2016) 45:e391–8. 10.1016/j.lpm.2016.10.003 27816343

[6] Wang R Langhammer B. Predictors of quality of life for chronic stroke survivors in relation to cultural differences: a literature review. *Scand J Caring Sci.* (2018) 32:502–14. 10.1111/scs.12533 28949412

[7] Elamy AH Shuaib A Carriere KC Jeerakathil T. Common comorbidities of stroke in the Canadian population. *Can J Neurol Sci.* (2020) 47:314–9. 10.1017/cjn.2020.17 31955718

[8] Gallacher KI Jani BD Hanlon P Nicholl BI Mair FS. Multimorbidity in stroke. *Stroke.* (2019) 50:1919–26. 10.1161/strokeaha.118.020376 31233391

[9] Fridman S Bres Bullrich M Jimenez-Ruiz A Costantini P Shah P Just C Stroke risk, phenotypes, and death in COVID-19: systematic review and newly reported cases. *Neurology.* (2020) 95:e3373–85. 10.1212/wnl.0000000000010851 32934172

[10] Veldema J Jansen P. Resistance training in stroke rehabilitation: systematic review and meta-analysis. *Clin Rehabil.* (2020) 34:1173–97. 10.1177/0269215520932964 32527148

[11] Nelson MLA McKellar KA Yi J Kelloway L Munce S Cott C Stroke rehabilitation evidence and comorbidity: a systematic scoping review of randomized controlled trials. *Top Stroke Rehabil.* (2017) 24:374–80. 10.1080/10749357.2017.1282412 28218020

[12] World Health Organization [WHO]. *Noncommunicable Diseases.* (2021). Available online at: https://www.who.int/news-room/fact-sheets/detail/noncommunicable-diseases (accessed November 12, 2021).

[13] Chiavaroli L Viguiliouk E Nishi SK Blanco Mejia S Raheliæ D Kahleová H DASH dietary pattern and cardiometabolic outcomes: an umbrella review of systematic reviews and meta-analyses. *Nutrients.* (2019) 11:338. 10.3390/nu11020338 30764511 PMC6413235

[14] Lim H Choue R. Impact of nutritional status and dietary quality on stroke: do we need specific recommendations? *Eur J Clin Nutr.* (2013) 67:548–54. 10.1038/ejcn.2013.30 23443833

[15] Wirt A Collins CE. Diet quality–what is it and does it matter? *Public Health Nutr.* (2009) 12:2473–92. 10.1017/s136898000900531x 19335941

[16] Chiba R Tominaga S Mikami K Kitajima M Urushizaka M Tomisawa T Factors influencing quality of life in patients with stroke: focus on eating habits. *J Stroke Cerebrovasc Dis.* (2019) 28:1623–8. 10.1016/j.jstrokecerebrovasdis.2019.02.031 30902395

[17] SakaiKKinoshitaSTsuboiMFukuiRMomosakiRWakabayashiH. Effects of nutrition therapy in older patients with stroke undergoing rehabilitation: a systematic review and meta-analysis. *J Nutr Health Aging.* (2019) 23:21–6. 10.1007/s12603-018-1095-4 30569064

[18] World Health Organization [WHO]. *International Classification of Disease 10th Revision (ICD-10).* (2019). Available online at: https://icd.who.int/browse10/2019/en#I60-I69 (accessed September 10, 2021).

[19] Faul F Erdfelder E Lang AG Buchner A. G*Power 3: a flexible statistical power analysis program for the social, behavioral, and biomedical sciences. *Behav Res Methods.* (2007) 39:175–91. 10.3758/bf03193146 17695343

[20] Ministry of Health [MOH]. *Coronavirus Disease (COVID-19) Outbreak in Vietnam.* (2020). Available online at: https://ncov.moh.gov.vn/ (accessed May 5, 2020).

[21] World Health Organization [WHO]. *Country & Technical Guidance-Coronavirus Disease (COVID-19).* (2020). Available online at: https://www.who.int/emergencies/diseases/novel-coronavirus-2019/technical-guidance (accessed March 10, 2020).

[22] Quan H Li B Couris CM Fushimi K Graham P Hider P Updating and validating the Charlson comorbidity index and score for risk adjustment in hospital discharge abstracts using data from 6 countries. *Am J Epidemiol.* (2011) 173:676–82. 10.1093/aje/kwq433 21330339

[23] Charlson ME Pompei P Ales KL MacKenzie CR. A new method of classifying prognostic comorbidity in longitudinal studies: development and validation. *J Chronic Dis.* (1987) 40:373–83. 10.1016/0021-9681(87)90171-8 3558716

[24] Warren-Findlow J Reeve CL Racine EF. Psychometric validation of a brief self-report measure of diet quality: the DASH-Q. *J Nutr Educ Behav.* (2017) 49:92–9.e1. 10.1016/j.jneb.2016.09.004 27818038

[25] Nguyen LTK Do BN Vu DN Pham KM Vu MT Nguyen HC Physical activity and diet quality modify the association between comorbidity and disability among stroke patients. *Nutrients.* (2021) 13:1641. 10.3390/nu13051641 34068135 PMC8152968

[26] Hays RD Morales LS. The RAND-36 measure of health-related quality of life. *Ann Med.* (2001) 33:350–7. 10.3109/07853890109002089 11491194

[27] Ngo-Metzger Q Sorkin DH Mangione CM Gandek B Hays RD. Evaluating the SF-36 health survey (version 2) in older Vietnamese Americans. *J Aging Health.* (2008) 20:420–36. 10.1177/0898264308315855 18381886 PMC4183463

[28] Nguyen HC Nguyen MH Do BN Tran CQ Nguyen TTP Pham KM People with suspected COVID-19 symptoms were more likely depressed and had lower health-related quality of life: the potential benefit of health literacy. *J Clin Med.* (2020) 9:965. 10.3390/jcm9040965 32244415 PMC7231234

[29] Nguyen DT Dang TC Nguyen QA Le TD Hoang TD Tran TNT The effect of subcutaneous injection of methylprednisolone acetate and lidocaine for refractory postherpetic neuralgia: a prospective, observational study. *Health Sci Rep.* (2021) 4:e271. 10.1002/hsr2.271 33855194 PMC8031000

[30] Hays RD Kallich J Mapes D Coons S Amin N Carter W *Kidney Disease Quality of Life Short Form (KDQOL-SF), Version 1.3: a Manual for Use and Scoring.* Santa Monica, CA: RAND Corporation (1997).

[31] Craig CL Marshall AL Sjöström M Bauman AE Booth ML Ainsworth BE International physical activity questionnaire: 12-country reliability and validity. *Med Sci Sports Exerc.* (2003) 35:1381–95. 10.1249/01.Mss.0000078924.61453.Fb 12900694

[32] Pham T Bui L Nguyen A Nguyen B Tran P Vu P The prevalence of depression and associated risk factors among medical students: an untold story in Vietnam. *PLoS One.* (2019) 14:e0221432. 10.1371/journal.pone.0221432 31430339 PMC6701769

[33] Lee PH Macfarlane DJ Lam TH Stewart SM. Validity of the international physical activity questionnaire short form (IPAQ-SF): a systematic review. *Int J Behav Nutr Phys Act.* (2011) 8:115. 10.1186/1479-5868-8-115 22018588 PMC3214824

[34] World Health Organization [WHO]. *WHO Disability Assessment Schedule 2.0 (WHODAS 2.0).* (2010). Available online at: https://www.who.int/classifications/icf/whodasii/en/ (accessed September 12, 2021).

[35] Sørensen K Van den Broucke S Fullam J Doyle G Pelikan J Slonska Z Health literacy and public health: a systematic review and integration of definitions and models. *BMC Public Health.* (2012) 12:80. 10.1186/1471-2458-12-80 22276600 PMC3292515

[36] Do BN Nguyen PA Pham KM Nguyen HC Nguyen MH Tran CQ Determinants of health literacy and its associations with health-related behaviors, depression among the older people with and without suspected COVID-19 symptoms: a multi-institutional study. *Front Public Health.* (2020) 8:581746. 10.3389/fpubh.2020.581746 33313037 PMC7703185

[37] Duong TV Nguyen TTP Pham KM Nguyen KT Giap MH Tran TDX Validation of the short-form health literacy questionnaire (HLS-SF12) and its determinants among people living in rural areas in Vietnam. *Int J Environ Res Public Health.* (2019) 16:3346. 10.3390/ijerph16183346 31514271 PMC6765800

[38] Tsartsalis D Dragioti E Kontoangelos K Pitsavos C Sakkas P Papadimitriou GN The impact of depression and cardiophobia on quality of life in patients with essential hypertension. *Psychiatriki.* (2016) 27:192–203. 10.22365/jpsych.2016.273.192 27837573

[39] Witassek F Springer A Adam L Aeschbacher S Beer JH Blum S Health-related quality of life in patients with atrial fibrillation: the role of symptoms, comorbidities, and the type of atrial fibrillation. *PLoS One.* (2019) 14:e0226730. 10.1371/journal.pone.0226730 31869399 PMC6927649

[40] Zemed A Nigussie Chala K Azeze Eriku G Yalew Aschalew A. Health-related quality of life and associated factors among patients with stroke at tertiary level hospitals in Ethiopia. *PLoS One.* (2021) 16:e0248481. 10.1371/journal.pone.0248481 33735246 PMC7971497

[41] Candelario-Jalil E Paul S. Impact of aging and comorbidities on ischemic stroke outcomes in preclinical animal models: a translational perspective. *Exp Neurol.* (2021) 335:113494. 10.1016/j.expneurol.2020.113494 33035516 PMC7874968

[42] Cipolla MJ Liebeskind DS Chan S-L. The importance of comorbidities in ischemic stroke: impact of hypertension on the cerebral circulation. *J Cereb Blood Flow Metab.* (2018) 38:2129–49. 10.1177/0271678x18800589 30198826 PMC6282213

[43] Levard D Buendia I Lanquetin A Glavan M Vivien D Rubio M. Filling the gaps on stroke research: focus on inflammation and immunity. *Brain Behav Immun.* (2021) 91:649–67. 10.1016/j.bbi.2020.09.025 33017613 PMC7531595

[44] Cho S Yang J. What do experimental models teach us about comorbidities in stroke? *Stroke.* (2018) 49:501–7. 10.1161/strokeaha.117.017793 29311269 PMC5780236

[45] Roca F Lang PO Chassagne P. Chronic neurological disorders and related comorbidities: role of age-associated physiological changes. *Handb Clin Neurol.* (2019) 167:105–22. 10.1016/b978-0-12-804766-8.00007-8 31753128

[46] Gorelick PB Furie KL Iadecola C Smith EE Waddy SP Lloyd-Jones DM Defining optimal brain health in adults: a presidential advisory from the American heart association/American stroke association. *Stroke.* (2017) 48:e284–303. 10.1161/str.0000000000000148 28883125 PMC5654545

[47] She R Yan Z Hao Y Zhang Z Du Y Liang Y Health-related quality of life after first-ever acute ischemic stroke: associations with cardiovascular health metrics. *Qual Life Res.* (2021) 30:2907–17. 10.1007/s11136-021-02853-x 33932220

[48] Pisegna J Xu M Spees C Krok-Schoen JL. Mental health-related quality of life is associated with diet quality among survivors of breast cancer. *Support Care Cancer.* (2021) 29:2021–8. 10.1007/s00520-020-05698-1 32844314

[49] XuFCohenSALofgrenIEGreeneGWDelmonicoMJGreaneyML. Relationship between diet quality, physical activity and health-related quality of life in older adults: findings from 2007-2014 national health and nutrition examination survey. *J Nutr Health Aging.* (2018) 22:1072–9. 10.1007/s12603-018-1050-4 30379305

[50] Knorr D Khoo CH. COVID-19 and food: challenges and research needs. *Front Nutr.* (2020) 7:598913. 10.3389/fnut.2020.598913 33344494 PMC7744420

