# Supplementary material for: Negative Impact of Comorbidity on Health-Related Quality of Life Among Patients With Stroke as Modified by Good Diet Quality
Source: Front Med (Lausanne). 2022 May 6;9:836027. doi: 10.3389/fmed.2022.836027 (PMC9121115; doi:10.3389/fmed.2022.836027)
Supplement: Supplementary file 1 [file Table_1.DOCX]

Negative Impact of Comorbidity on Health-Related Quality of Life among Stroke Patients as Modified by Good Diet Quality

**Table S1**. Spearman’s correlations (rho) among the studied variables (n= 951).

| Variables | Age | Gender | Marital status | Occupation | Stroke occurrence | Stroke classification | CCI | Drinking | MET-min/wk | DASH-Q | WHODAS 2.0 |
| --- | --- | --- | --- | --- | --- | --- | --- | --- | --- | --- | --- |
| Gender | -0.09 |  |  |  |  |  |  |  |  |  |  |
| Marital status | 0.12 | -0.23 |  |  |  |  |  |  |  |  |  |
| Occupation | **0.35** | -0.02 | 0.09 |  |  |  |  |  |  |  |  |
| Stroke occurrence | 0.08 | 0.07 | 0.00 | 0.08 |  |  |  |  |  |  |  |
| Stroke classification | -0.20 | 0.02 | -0.05 | -0.11 | -0.10 |  |  |  |  |  |  |
| CCI | 0.08 | -0.02 | -0.11 | 0.11 | 0.12 | -0.09 |  |  |  |  |  |
| Drinking | -0.13 | **0.45** | -0.10 | -0.10 | -0.01 | 0.02 | 0.02 |  |  |  |  |
| MET-min/wk | -0.17 | 0.09 | -0.02 | **-0.32** | -0.11 | 0.06 | -0.18 | 0.19 |  |  |  |
| DASH-Q | -0.06 | -0.01 | 0.07 | -0.18 | -0.13 | 0.04 | -0.15 | 0.04 | 0.46 |  |  |
| WHODAS 2.0 | 0.12 | -0.07 | 0.08 | 0.22 | 0.03 | 0.01 | **0.30** | -0.06 | **-0.32** | -0.21 |  |
| HL index | -0.22 | 0.13 | -0.23 | -0.09 | -0.02 | 0.04 | 0.07 | 0.23 | 0.13 | 0.09 | -0.17 |

Abbreviations: CCI, Charlson Comorbidity Index; MET-min/wk, metabolic equivalent task scored in minutes per week; DASH-Q, Dietary Approaches to Stop Hypertension Quality; WHODAS 2.0, World Health Organization Disability Assessment Schedule 2.0; HL, health literacy.
